# Muscular Response in Sleep-Disordered Breathing Patients: New Methods of Assessment and Measurement

**DOI:** 10.3390/clockssleep8040065

**Published:** 2026-10-07

**Authors:** Ruggero Campisi, Ilaria Falco, Giannicola Iannella, Antonio Maniaci, Miriam Nava, Gianluca Bronzetti, Davide Bianchi, Alice Cescutti, Manuele Casale, Antonio Moffa, Chiara Bianchini, Stefano Pelucchi, Pietro Bonzagni, Kenny Peter Pang, Mario Pierri, Claudio Vicini

**Affiliations:** 1 GVM Care & Research, San Pier Damiano Hospital, Via Portisano 1, 48018 Faenza, Italy; 2 Department of Sense Organs, Sapienza University of Rome, Piazzale Aldo Moro 5, 00185 Rome, Italy; 3 Department of Otolaryngology, Kore University of Enna, 94100 Enna, Italy; 4 MedicAir Healthcare, Via Monte Rosa 61, 20149 Milan, Italy; 5 Integrated Therapies in Otolaryngology, Fondazione Policlinico Universitario Campus Bio-Medico, Via Alvaro del Portillo 200, 00128 Rome, Italy; 6 Department of Otolaryngology, University of Ferrara, 44121 Ferrara, Italy; 7 Asia Sleep Centre, Paragon Medical Centre, 290 Orchard Road, #18-04, Singapore 238859, Singapore; 8 Professional Master’s Programme in Dental Sleep Medicine, Alma Mater Studiorum University of Bologna, Via Zamboni 33, 40126 Bologna, Italy

**Keywords:** obstructive sleep apnea, REM sleep, NREM sleep, apnea-hypopnea index (AHI), neuromuscular response, clinical surrogate, endotyping, precision medicine

## Abstract

Background: Obstructive sleep apnea (OSA) is a heterogeneous disorder influenced by both anatomical collapsibility and non-anatomical factors, such as neuromuscular response. Identifying these traits through simplified clinical surrogates is crucial for personalized precision medicine. Methods: This exploratory retrospective study evaluated 207 home polygraphy recordings of Italian patients to assess sleep-stage-specific Apnea–Hypopnea Index (REM vs. NREM AHI) as a clinical surrogate indicator of the balance between anatomical collapsibility and neuromuscular compensation. Patients were stratified by severity into simple snorers (AHI < 5 events/h), mild OSA (AHI 5–15 events/h), and severe OSA (AHI > 30 events/h). REM and NREM AHI values were compared and correlated with Apnea Index (AI), Hypopnea Index (HI), body mass index (BMI), and cumulative nocturnal desaturation indices (CT < 90% and T < 90 min). Results: In non-severe patients (simple snorers and mild OSA), REM AHI was significantly higher than NREM AHI (REM/NREM ratio > 1), suggesting relative neuromuscular protection during NREM sleep. Conversely, in severe OSA patients, the REM/NREM ratio progressively decreased and approached unity, with NREM AHI prevailing. Furthermore, NREM predominance was significantly associated with a higher AI and a worse cumulative desaturation indicating a globally more severe disease. Conclusions: A predominance of REM AHI characterizes milder OSA presentations with effective NREM muscular compensation, whereas NREM predominance aligns with an anatomically dominant, severe phenotype. Decomposing AHI into REM and NREM components provides a practical, readily available clinical surrogate to estimate the anatomy–neuromuscular balance and guide PALM-oriented personalized treatments.

## 1. Introduction

Obstructive sleep apnea (OSA) is a highly prevalent chronic disorder affecting hundreds of millions of adults worldwide, carrying substantial cardiovascular, metabolic, and neurocognitive consequences [1,2]. While anatomical narrowing of the upper airway is a primary prerequisite for pharyngeal collapse, anatomy alone fails to explain the marked clinical heterogeneity of the disease [3,4]. Landmark physiological studies have demonstrated that non-anatomical endotypic traits critically interact with structural vulnerability to dictate disease expression [5,6].

### 1.1. Purpose of the Study

This exploratory retrospective study aims to critically evaluate the role of sleep-stage-specific AHI (REM vs. non-REM) as a clinical “surrogate”, indicator of the balance between anatomical collapsibility and neuromuscular compensation in OSA, with the goal of promoting qualitative phenotyping useful for precision medicine. To this end, it analyzes 207 home polygraphy recordings (Nox Medical, Nox BodySleep™ algorithm) of Italian patients with desaturation events, comparing AHI-REM and AHI-NREM across different severity levels (simple snorers, mild OSA, severe OSA) and correlating these measures with cumulative nocturnal desaturation indices (CT < 90% and T < 90 min), Apnea Index (AI), Hypopnea Index (HI), and BMI. The operational objective is to determine whether REM or NREM predominance can serve as a rapid clinical signal of the “anatomy vs. neuromuscular response” balance (PALM scheme) and thus guide more targeted therapeutic choices beyond the sole metric of total AHI.

### 1.2. Pathogenesis

Under the qualitative phenotyping model proposed by Bosi et al. [6], OSA pathogenesis is governed by passive pharyngeal collapsibility in dynamic equilibrium with three primary non-anatomical pathophysiological traits: the respiratory arousal threshold (AT), ventilatory control instability (loop gain, LG), and upper airway muscle responsiveness (muscular gain, UAG).

**Figure 1:**
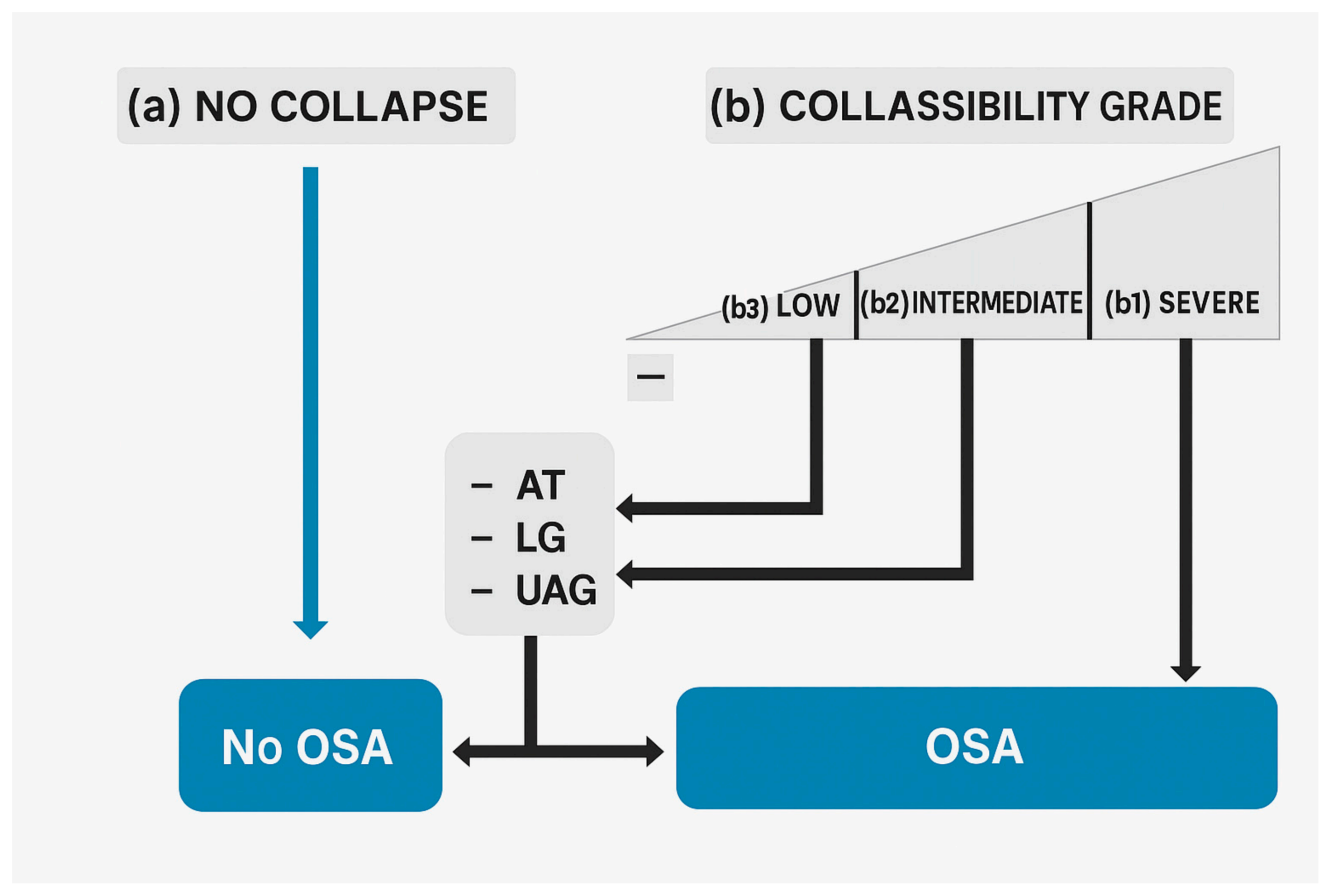
Adapted from Bosi et al. [6]. (**a**) Individuals without any degree of UA collapsibility do not develop OSA, even if other non-anatomical pathophysiological traits (PTs) are abnormal. (**b1**) Patients with severe UA collapsibility develop OSA regardless of any other non-anatomical PT. (**b2**) Patients with an intermediate degree of UA collapsibility develop OSA depending on the pathological severity of other non-anatomical PTs (AT, LG, and UAG) or on the degree of UA collapsibility itself. (**b3**) Patients with low UA collapsibility may or may not develop OSA, but only in the presence of significant non-anatomical PTs.

The various conditions influencing the likelihood of developing OSA are summarized in the well-known Figure 1 from the article, which follows.

From this insightful article, an additional clinical surrogate emerges, useful for profiling OSA patients and introducing a clinical reasoning approach based on the PALM classification, where “*P*” stands for Pcrit, “*A*” for AT, “*L*” for LG, and “*M*” for muscle compensation.

This system classifies OSA patients into three subgroups based on the characterization of PTs. The PALM subgroups are as follows [6]:

- PALM 1: This group comprises approximately 23% of OSA patients and is characterized by high anatomical collapsibility (Pcrit greater than +2.5 cmH_2_O). Weight loss, positional therapy, oral appliances (OA), CPAP, and upper airway surgery are first-line treatments, as these therapies target anatomical factors (anatomical treatments).
- PALM 2: This is the largest subgroup, involving about 57% of OSA patients; it is characterized by intermediate collapsibility (Pcrit between +2.5 and –2.5 cmH_2_O). These patients are potential candidates for anatomical treatments (subgroup 2a) or for a combination of anatomical and non-anatomical therapies.
- PALM 3: This subgroup includes approximately 19% of OSA patients with low collapsibility (Pcrit less than –2.5 cmH_2_O), associated with abnormal non-anatomical PTs. These patients are potential candidates for therapeutic options such as weight loss, oral appliances (OA), oxygen, and drugs targeting loop gain or arousal threshold.

**Figure 2:**
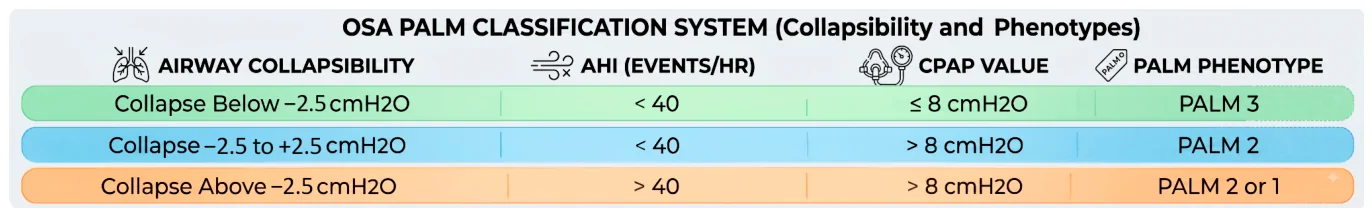
Adapted from Bosi et al. [6].

This conceptual framework underpins the PALM classification scheme (Pcrit, Arousal threshold, Loop gain, Muscle responsiveness), which stratifies patients according to anatomical collapsibility and identifies candidates for targeted non-CPAP therapies [6,7]. To overcome the need for complex, invasive laboratory procedures, pragmatic clinical “surrogates” have been proposed to estimate these traits [6,8]. For instance, combining diagnostic polygraphy metrics with therapeutic CPAP levels provides a readily accessible surrogate to delineate PALM phenotypes in daily practice (Figure 2).

Among the non-anatomical traits, upper airway muscle response represents a vital endogenous defense mechanism [5,9]. Pharyngeal dilator muscles—predominantly the genioglossus (GG) and tensor veli palatini—exhibit marked state-dependent modulation [9,10]. During wakefulness and NREM sleep, elevated neuromuscular tone can actively compensate for an anatomically crowded lumen [10,11]. Conversely, rapid eye movement (REM) sleep is characterized by profound physiological muscle atonia mediated by pontine premotor inhibition of cranial motor neurons [12,13]. Deprived of active neuromuscular compensation, upper airway stability decreases significantly, leading to state-dependent collapse [14,15].

In an effort to quantify this compensatory capacity without invasive intramuscular electrodes, Li et al. proposed using the disparity between AHI during REM and NREM sleep as a clinical surrogate for muscle responsiveness, incorporating it into a predictive scoring model for surgical outcomes (Table 1) [16].

The clinical relevance of REM-related muscle vulnerability has been recognized since early sleep investigations. Pharmacological trials demonstrating REM suppression (e.g., via protriptyline) documented notable reductions in nocturnal desaturation despite persistent anatomical events, underscoring the protective role of preserving non-atonic sleep states (Table 2) [17].

Similarly, Cartwright et al. demonstrated that while sleep posture exerts a substantial mechanical influence, REM sleep consistently amplifies apnea frequency across body positions compared to NREM sleep (Table 3) [18].

**Figure 3:**
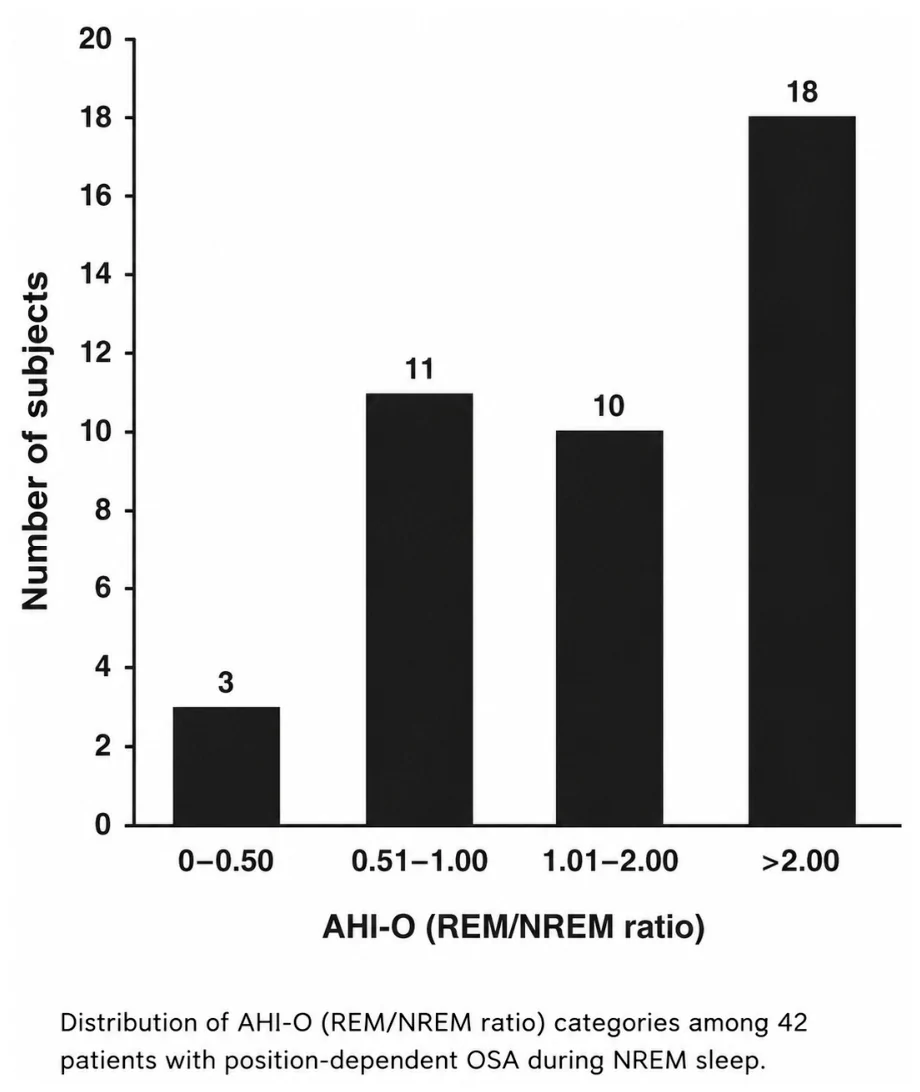
Adapted from Pevernagie et al. [19].

Furthermore, in clinical populations, Pevernagie et al. documented that a substantial proportion of patients exhibit an AHI during REM sleep that is more than double that observed during NREM sleep (Figure 3) [19].

More recent physiological investigations by Carberry and Eckert confirmed that pharyngeal collapsibility (Pcrit) is significantly higher during REM sleep compared to slow-wave and N2 sleep, mirroring the progressive decline in genioglossus EMG activity [14]. However, as highlighted by Messineo et al., REM sleep events are also influenced by central ventilatory drive withdrawal, indicating that the stage-dependent distribution of respiratory events reflects a multifactorial balance [15]. Furthermore, stage-dependent event distributions reflect complex interactions between anatomical vulnerability and non-anatomical control mechanisms. While early disease frequently manifests during REM sleep due to physiological muscle atonia, NREM-predominant patterns can emerge either through progressive structural anatomical collapse or via non-anatomical ventilatory instability, such as cyclic alternating pattern (CAP) oscillations and high loop gain [20].

From these observations emerges our working hypothesis: when upper airway neuromuscular compensation is functionally intact, it protects the airway during NREM sleep, confining obstructive events primarily to REM sleep (AHIREM > AHINREM) [16,19]. Conversely, when severe structural collapsibility overwhelms muscular capacity or when neuromuscular response fails, obstructive events occur pan-phasically across both sleep states, diminishing or reversing this ratio (AHINREM ≥ AHIREM) [20,21].

The operational purpose of this exploratory retrospective study is to evaluate the sleep-stage-specific AHI (REM vs. NREM) across different severity strata (simple snorers, mild OSA, and severe OSA) obtained via validated home respiratory polygraphy. Specifically, we assessed whether the REM/NREM AHI ratio can serve as a pragmatic, indirect clinical surrogate of the balance between anatomical collapsibility and neuromuscular compensation, examining its association with cumulative nocturnal hypoxemia and apnea severity.

## 2. Results

**Figure 4:**
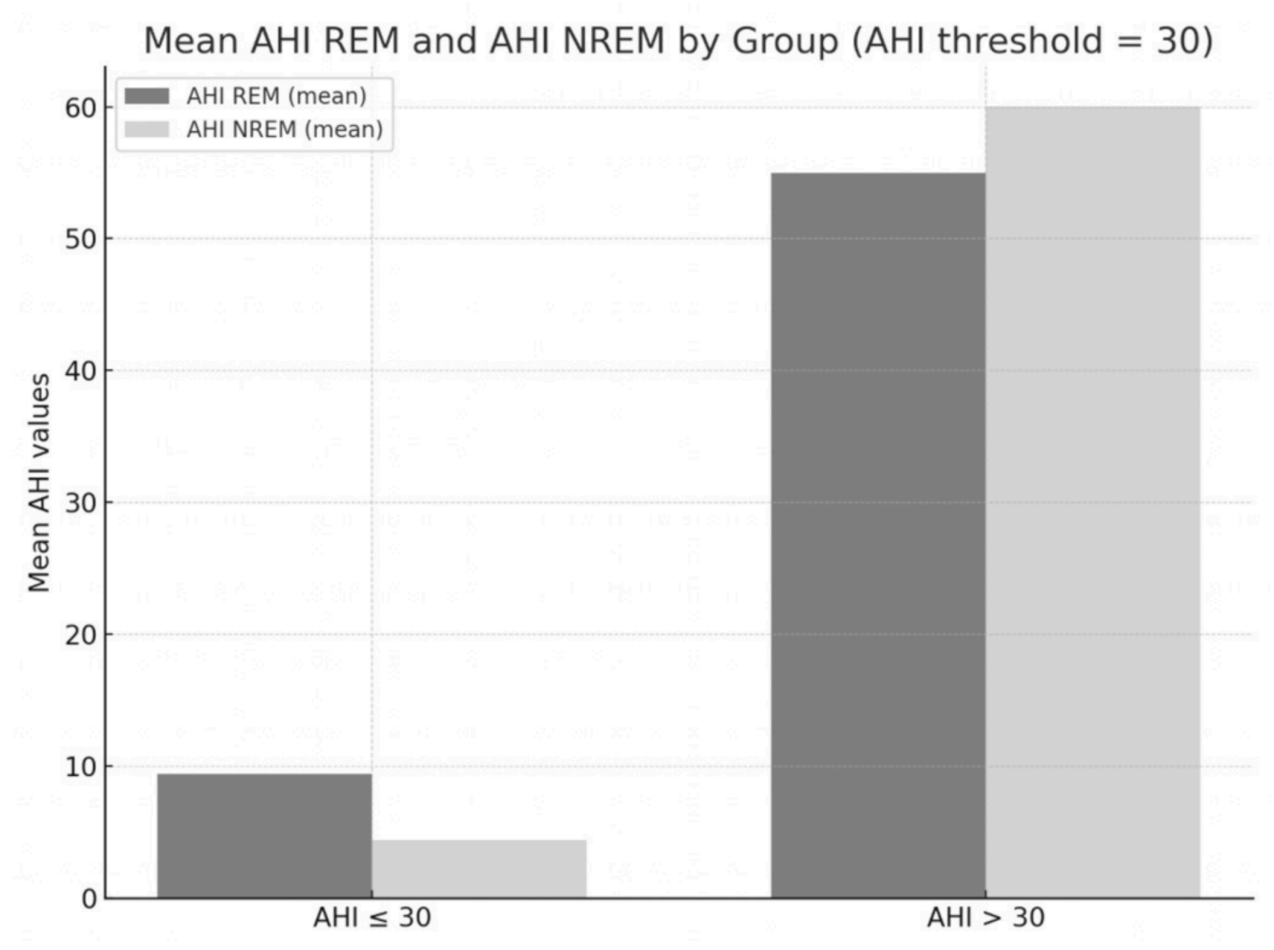
Graph of mean AHI-REM and AHI-NREM by groups (AHI threshold = 30).

Two populations: non-severe OSA group and severe OSA group (Table 4 and Figure 4). In the two patient populations, we observed ambivalent and contrasting data. In the non-severe OSA group, AHI tends to be higher during REM sleep compared to non-REM sleep, with a highly significant difference (*t*-test *p* = 8.8 × 10^−7^, Wilcoxon *p* = 3.1 × 10^−6^), with a mean AHI-REM of 9.4/h and a mean AHI-non-REM of 4.4/h (a difference of 5 events/h).

In the severe OSA group, the trend changes, and AHI tends to be higher during non-REM sleep, although with a significant but less marked difference (*t*-test *p* = 0.025, Wilcoxon *p* = 0.022). In this group, the mean AHI-REM is 54.9/h, while the mean AHI-non-REM is 60.1/h. Once again, there is a difference of 5.1 events/h, but in the opposite direction between the two sleep phases.

Regarding the first group, consisting of patients with non-severe OSA (101 patients), the tendency to have a higher AHI-REM, as reflected by the previously reported data, is observed in 66 patients, corresponding to 65% of the total. In this group, the predominance of severity in REM vs. non-REM does not markedly affect nocturnal desaturation parameters such as CT90%. Moreover, no significant correlations are evident with the Apnea Index (AI) or the Hypopnea Index (HI). BMI tends to be higher in the group with greater NREM predominance, but not in a statistically significant manner. In summary, in the group of non-severe apneics, the predominance of events in REM vs. NREM does not significantly influence AI, HI, or desaturation parameters.

**Figure 5:**
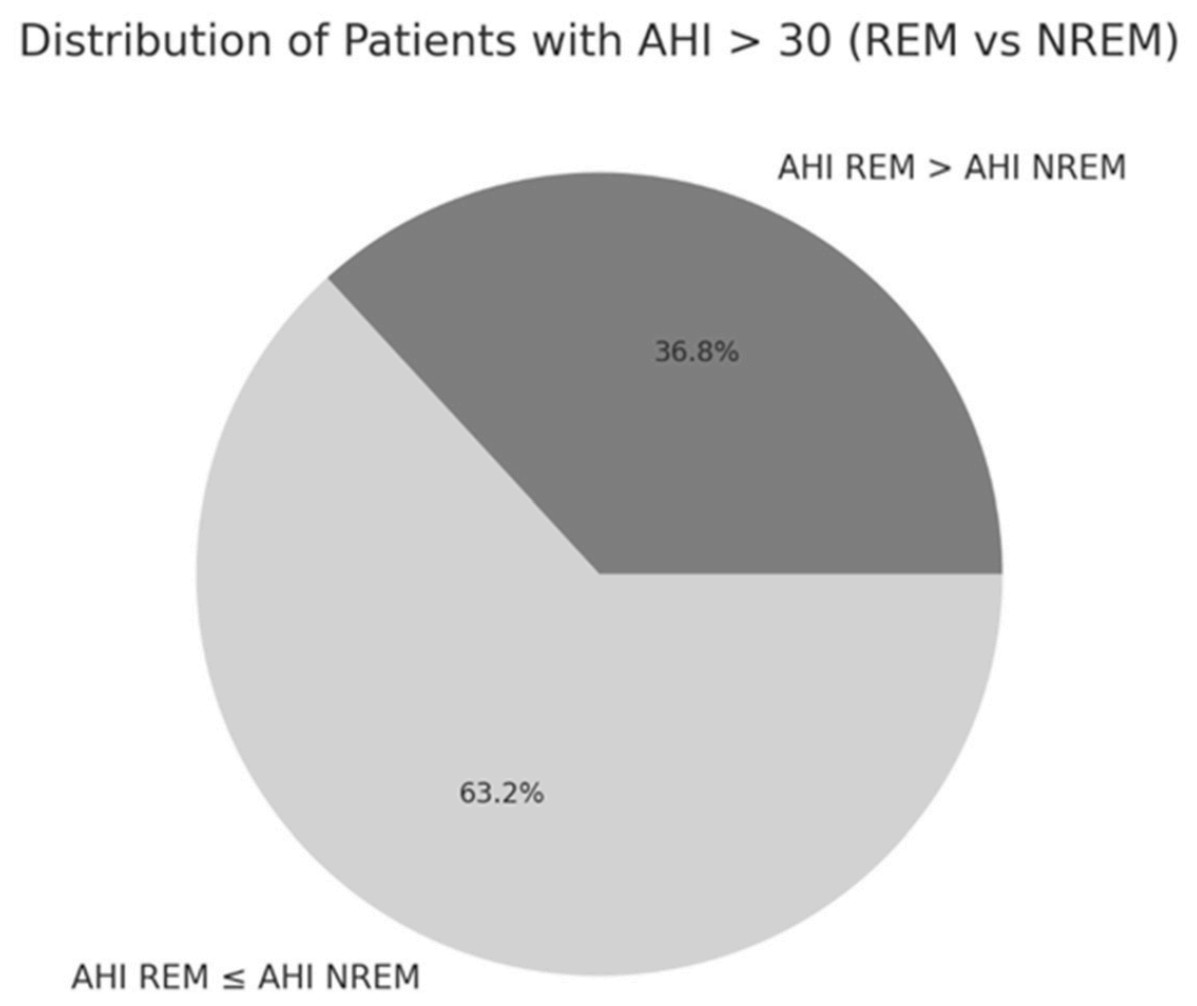
Distribution of AHI-REM vs. AHI-NREM in severe OSA.

Among the 106 patients with severe OSA, 67 have a higher AHI in non-REM, representing 63.2% of the total (Figure 5).

In the severe OSA group (AHI > 30 events/h), patients with NREM ≥ REM predominance (63.2% of cases) exhibited a significantly higher Apnea Index (AI: 36.4 ± 26.1 vs. 9.0 ± 12.4 events/h; Mann–Whitney U test, *p* < 0.001) and spent a significantly greater proportion of sleep time in cumulative desaturation (CT90%: 27.3 ± 22.4% vs. 10.7 ± 14.1%, *p* = 0.005; T < 90 min: *p* = 0.016). In contrast, Hypopnea Index (HI) and BMI did not differ significantly between the two subgroups. In a multivariable linear regression model adjusting for BMI, NREM AHI remained significantly associated with CT90% (β = 0.28, *p* = 0.012), while BMI accounted for substantial shared variance (β = 0.34, *p* < 0.001). This confirms that NREM predominance coexists with, rather than directly causes, worse cumulative desaturation within an anatomically vulnerable phenotype.

Now, we present the analyses without subdividing between the non-severe and severe OSA groups. Instead, we consider in absolute terms only the patients with predominant AHI in REM (105 patients, 50.7% of the total) and those with predominant AHI in non-REM (102 patients, 49.3% of the total). The group with predominant non-REM AHI demonstrates the most relevant characteristics, which are summarized below (and later in a comprehensive table):

- Higher total AHI compared to the other group.
- Pearson r = 0.39 (*p* < 1 × 10^−8^), Spearman r = 0.37 (*p* < 1 × 10^−7^), corresponding to 46.5/h vs. 21.37/h.
- Higher Apnea Index (AI) than the REM-predominant group.
- Pearson r ≥ 0.25, Spearman r = 0.40, specifically 36.36/h vs. 8.98/h.
- HI shows a weak, borderline correlation, not statistically significant for substantial differences.
- Nonetheless, the mean values are reported: for the NREM-predominant group, mean HI = 17.09/h; for the REM-predominant group, 12.26/h.
- More severe T < 90% and T < 90 min (Spearman r = 0.32–0.33), indicating that patients with NREM > REM tend to spend more time in desaturation. Specifically: 27.26% vs. 10.67%.

After adjusting for multiple comparisons using the Benjamini–Hochberg procedure (q < 0.05), total AHI, AI, and cumulative desaturation metrics remained significantly correlated with NREM predominance. However, the correlation coefficients were moderate (Spearman ρ = 0.31–0.40), confirming that the REM/NREM disparity provides an indirect surrogate signal rather than an isolated, standalone predictor of hypoxemic severity.

**Figure 6:**
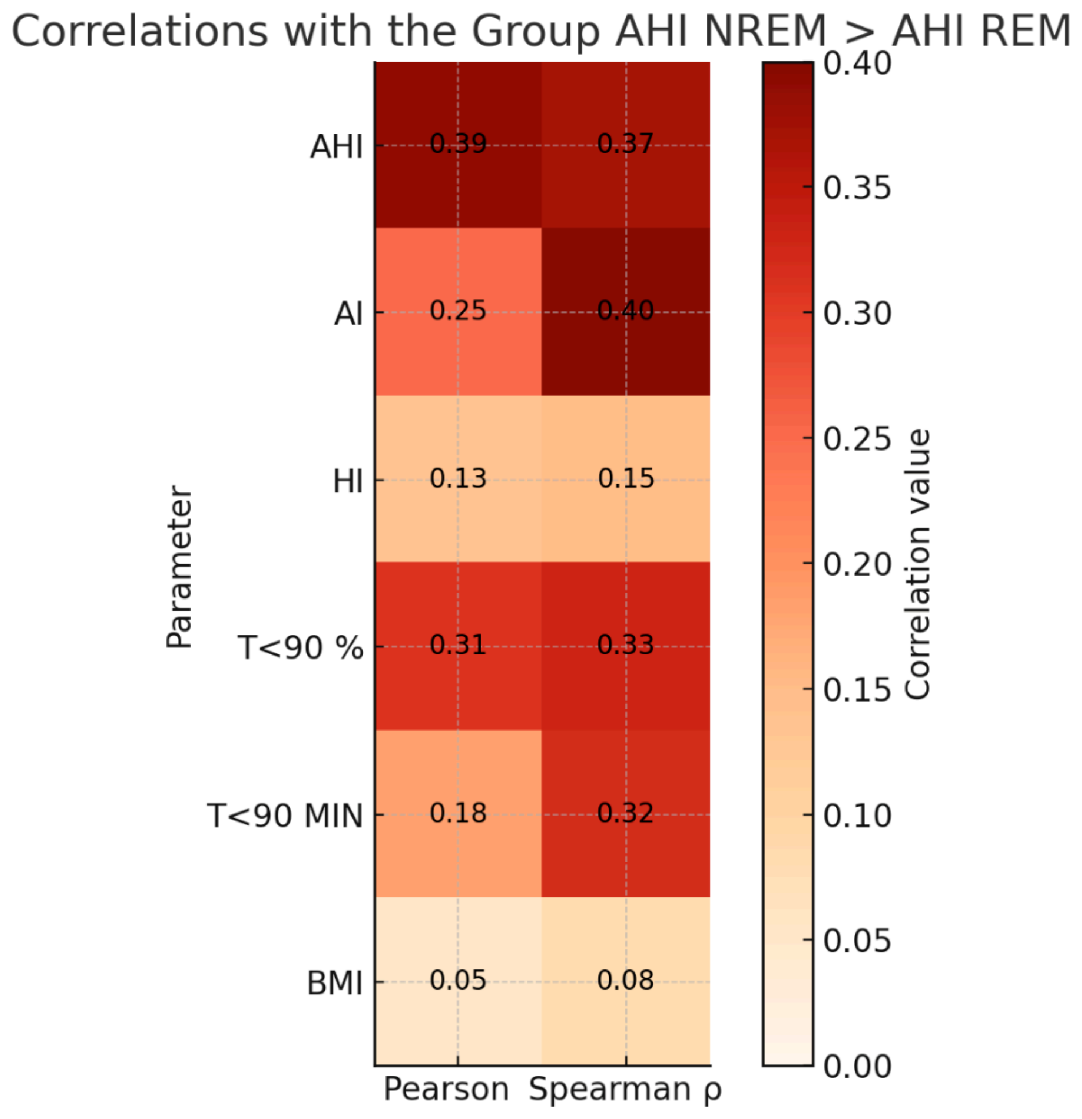
Correlation heatmap where the columns indicate the type of correlation (Pearson or Spearman). We represent clinical parameters, and the colors reflect the intensity and direction of the correlation. Total AHI, AI, and desaturation variables (T < 90%) show the strongest correlations with membership in the NREM > REM group.

The correlation heatmap is in Figure 6, below.

At this point, we stratified our cohort of 207 patients into the following categories:

- Simple snorers: AHI < 5 events/h.
- Mild OSA: AHI between 5 and 15 events/h.
- Severe OSA: AHI > 30 events/h.

We specify that in the examined cohort, no individuals met the criteria for moderate OSA (AHI between 15 and 30).

This three-tiered profiling, which we will now explore in detail, proved particularly insightful in providing arguments for the discussion that will follow. In particular, we focused on the REM and NREM AHI parameters, an analysis that yielded several noteworthy findings.

**Figure 7:**
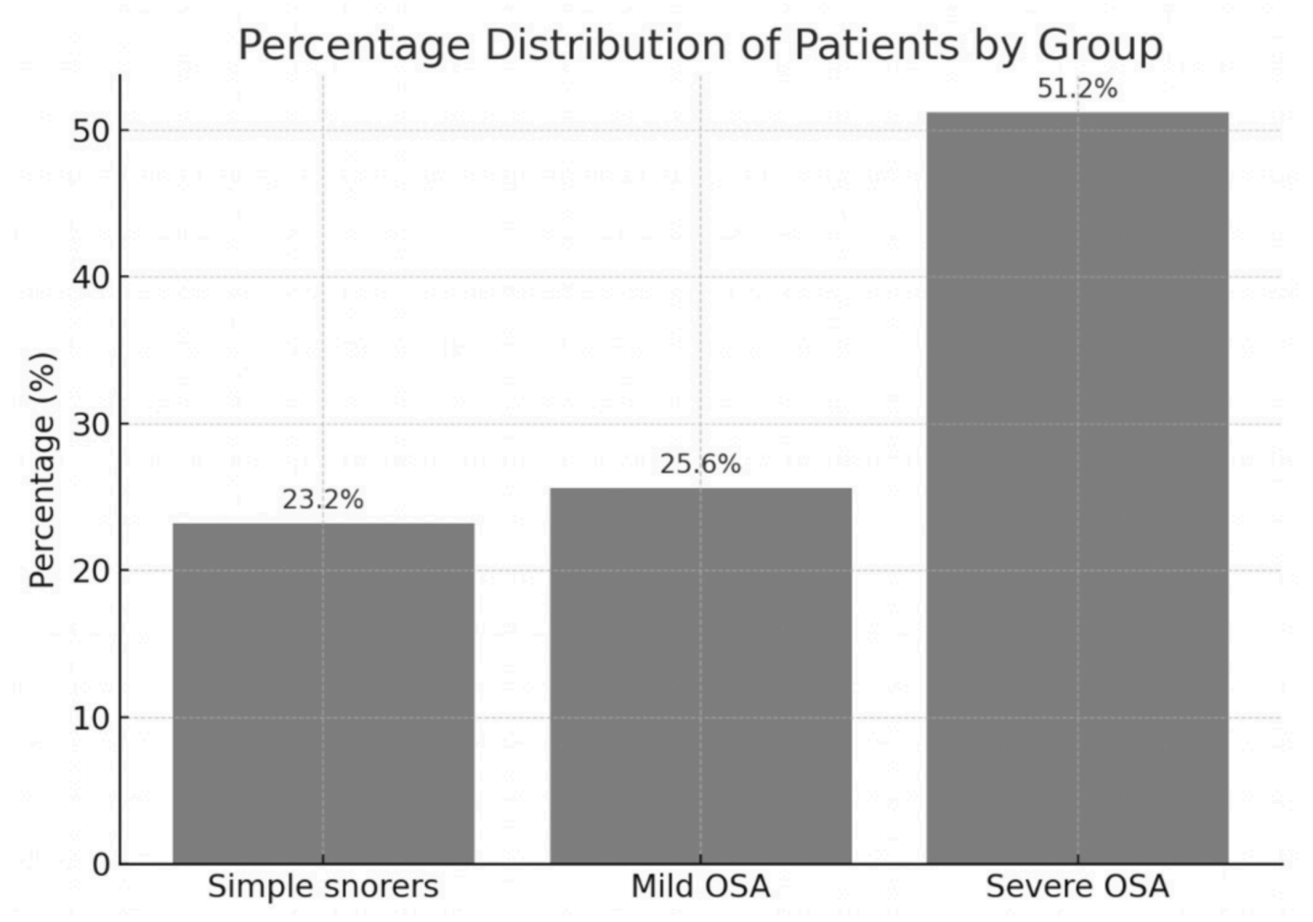
Percentage distribution of patients stratified by severity: simple snorers, mild OSA, and severe OSA.

This choice allows for a clear and distinct stratification of patients, without gray areas. Below is the distribution across the three groups, also illustrated graphically in Figure 7.

**Figure 8:**
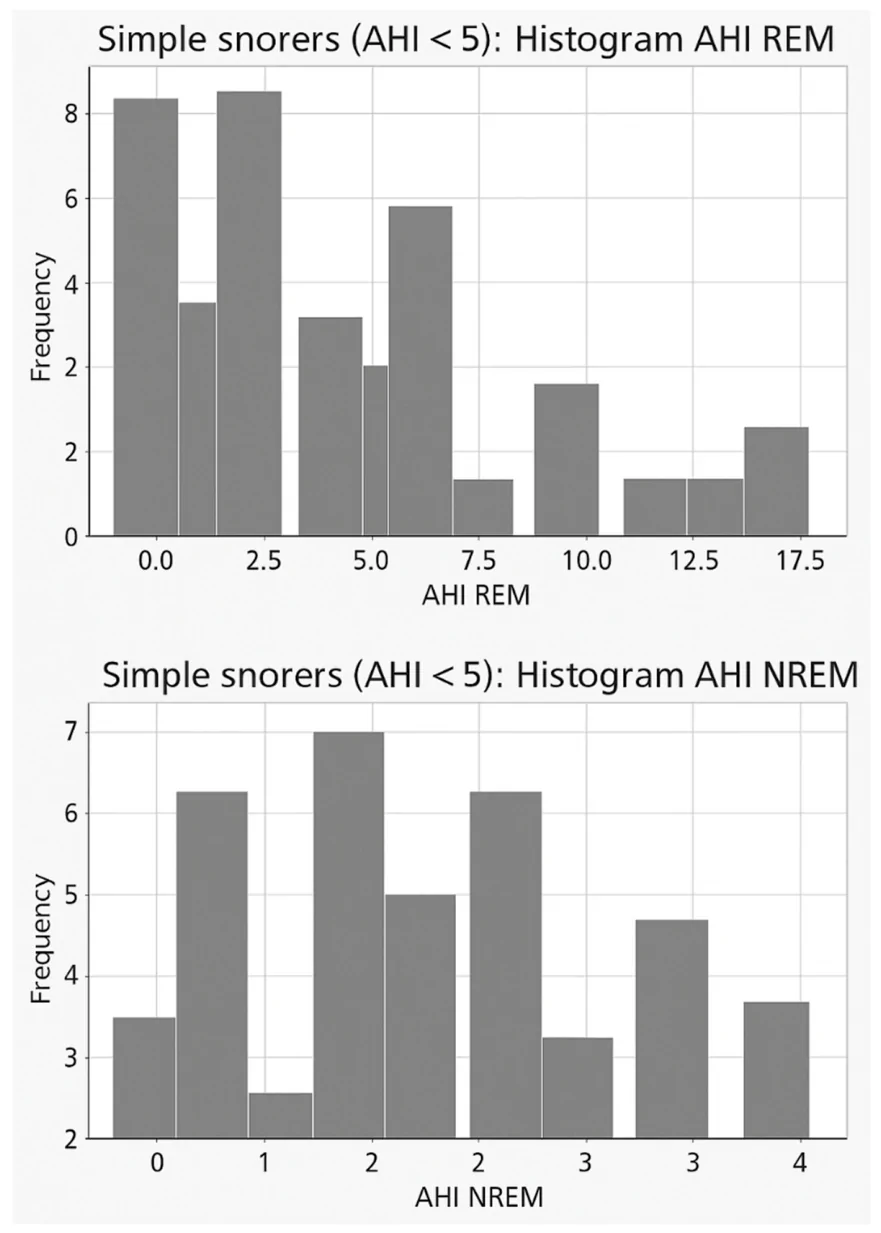
Histograms of REM AHI (**above**) and NREM AHI (**below**). Note the presence of REM AHI peaks above 15 events per hour, which are not observed in the NREM phase.

**Simple Snorers Subgroup (AHI < 5/h):** This subgroup includes 48 individuals (Table 5 and Table 6, Figure 8).

Table 5 shows the mean, median, standard deviation, maximum, and minimum values for the REM AHI and NREM AHI parameters. Notably, the severity indices expressed by the Apnea–Hypopnea Index are higher during the REM phase, at this stage.

**Mild OSA Subgroup (AHI between 5 and 15/h):** In this category, we have 53 patients (see distribution histogram above).

Table 7 shows the mean, median, standard deviation, maximum, and minimum values for the REM AHI and NREM AHI parameters. As in the previous subgroup, note that the severity indices expressed by the Apnea–Hypopnea Index are higher during the REM phase.

**Figure 9:**
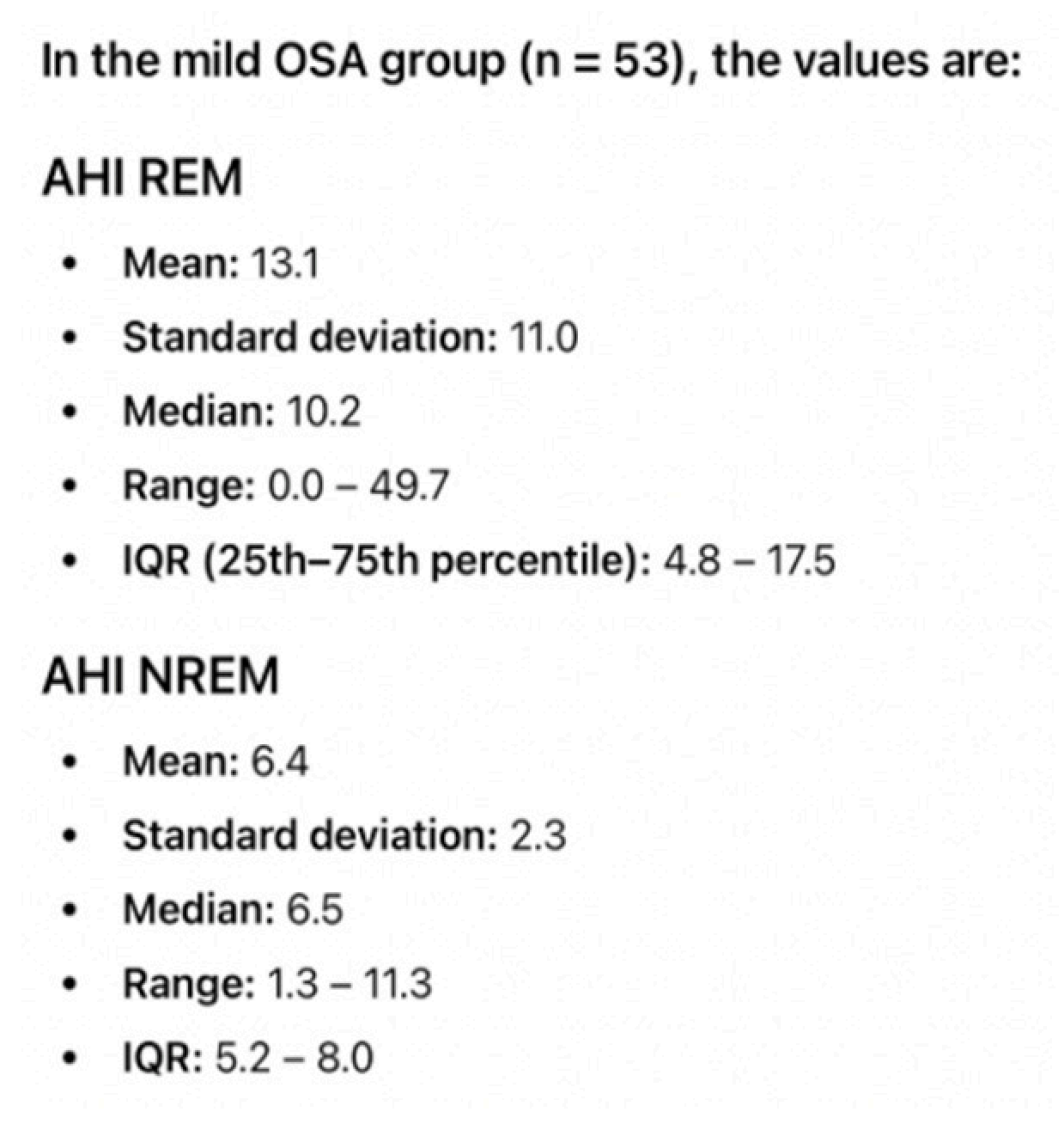
Mild OSA group, values and graph.

**Figure 10:**
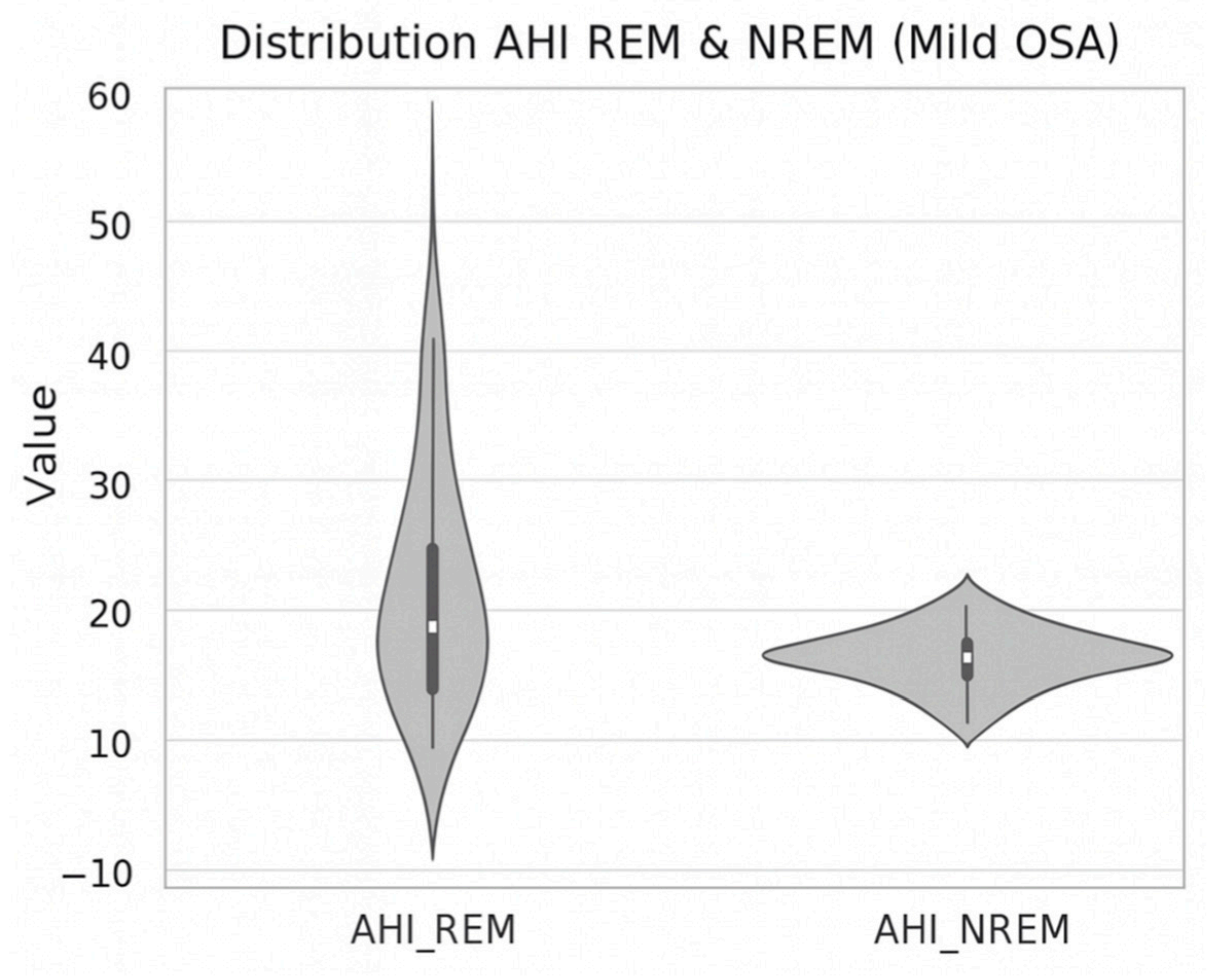
Mild OSA group, values and graph.

Mild OSA Group in Figure 9. The graphs also show that REM AHI dominates over NREM AHI in the mild OSA group. Specifically (Figure 10, right side), as a point of interest, REM AHI values are not only higher but also more variable. In contrast, NREM AHI appears more compact and clustered around consistently lower values.

**Figure 11:**
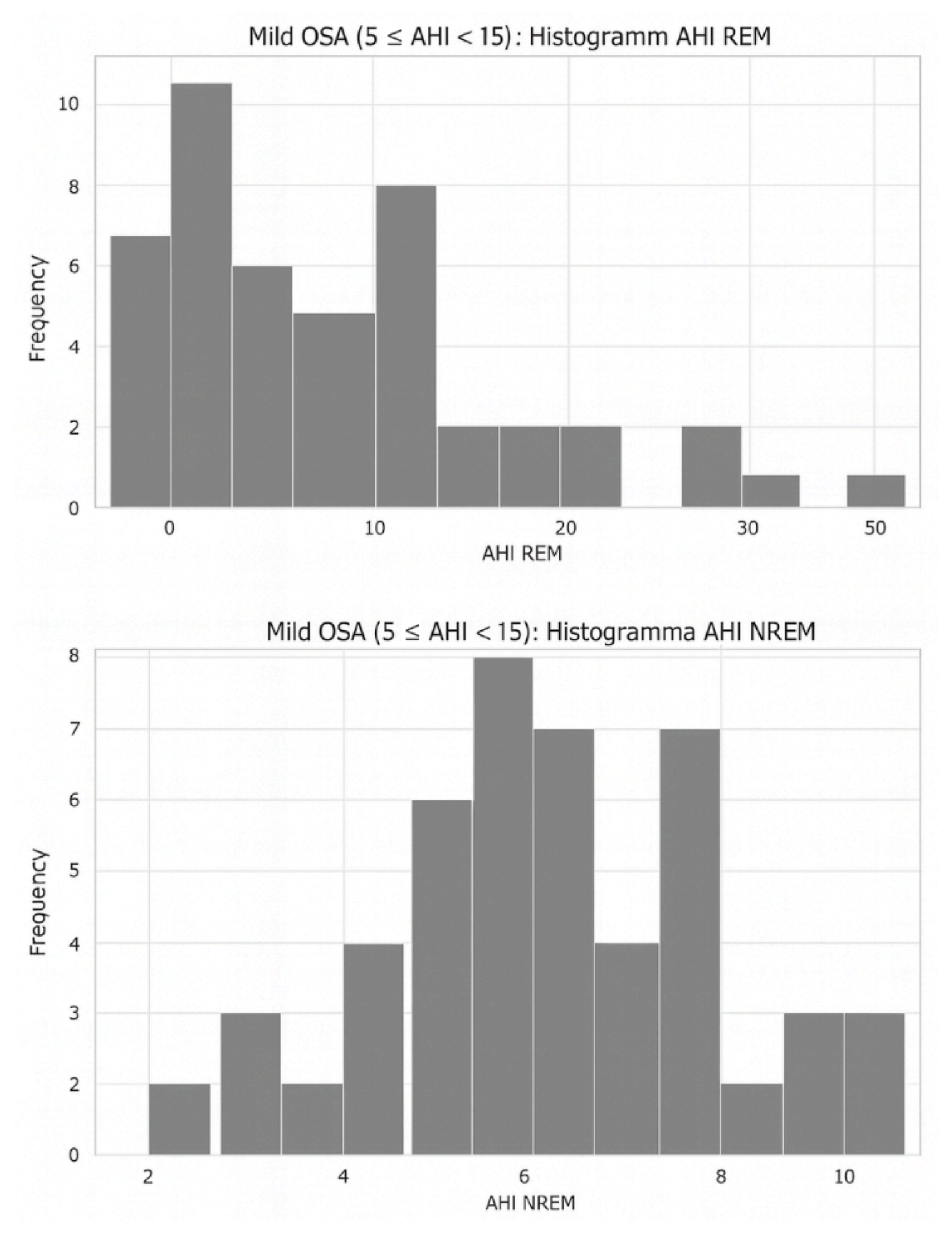
Histogram, mild OSA group.

Here as well, in Figure 11, we present the histogram showing the severity values distributed by frequency.

Here as well, from the histogram above, note that even very severe AHI values in REM are “normalized” during the NREM phase.

Statistical correlations—Mild OSA group.

### 2.1. Spearman Correlation Table Between AHI REM/AHI NREM and BMI, T < 90%, T < 90 min

Summary from Table 8:

- AHI REM shows a positive correlation with BMI (rho ≈ +0.23, *p* ≈ 0.10), but no correlation with desaturation parameters (T < 90%).
- AHI NREM shows a negative correlation with BMI (rho ≈ −0.27, *p* ≈ 0.05) and positive correlations with T < 90% (rho ≈ +0.23, *p* ≈ 0.10) and T < 90 min (rho ≈ +0.27, *p* ≈ 0.05).
- Correlations are generally weak to moderate, some at the threshold of statistical significance.

### 2.2. Severe OSA Subgroup (AHI > 30/h)

In this subgroup, we analyzed 106 patients (Table 9 and Table 10, Figure 5).

Table 9 shows the mean, median, standard deviation, maximum, and minimum values for the REM AHI and NREM AHI parameters. Notably, and in contrast to the other two previously examined subgroups, the severity indices expressed by the Apnea–Hypopnea Index are higher during the NREM phase. This applies both to the maximum values (see “max” in the table) and to the mean values. However, the median is slightly higher in the REM phase. Overall, the values in the two sleep stages remain very close.

The interpretation of the data, supported by Spearman correlation analysis (Table 11), allows us to conclude that desaturations (CT90%) are more strongly correlated with NREM AHI events than with REM AHI events (*p* ~ 0.59 vs. *p* ~ 0.33). Additionally, and quite intuitively, in patients with severe OSA, both REM AHI and NREM AHI tend to increase with increasing BMI (Table 11).

### 2.3. Calculation of a Delta for Disparity Between Rem and NREM AHI n Each Subgroup

At this point in the analysis, given the observation that REM AHI values are higher than NREM AHI in patients with non-severe OSA or in healthy individuals—as intuitively expected due to the muscular response that tends to “protect” the individual from sleep-related respiratory disturbances—but noting the loss of this behavior in patients with severe OSA, we considered it appropriate to calculate a delta (ratio) of difference between REM AHI and NREM AHI for each subgroup. This is the specific surrogate measure we propose for evaluating muscle response as a non-anatomical factor, and which we believe can serve as a reliable indicator of this trait.

This reasoning was driven by the observation that in the non-severe patient groups, both the maximum and mean REM AHI values were substantially higher compared to their NREM counterparts, whereas in the severe OSA group, this gap disappeared, resulting in mean and median values that were essentially comparable between REM AHI and NREM AHI.

This delta was calculated in different ways:

- **Mean of Ratios per Patient:** For each patient, we calculated the ratio of REM AHI/NREM AHI, in Equation (1), then computed the mean (ratio mean) within each group. This method respects intra-group variability and seems to us a more clinically relevant parameter, as it reflects the actual individual distribution (representing the average behavior of each patient).

(1) $$\mathrm{ratio}\_\mathrm{mean}=\frac{1}{n}\sum_{i=1}^{n}\frac{{\mathrm{AHI}\_\mathrm{REM}}_{i}}{{\mathrm{AHI}\_\mathrm{NREM}}_{i}}$$

Equation (1) ratio mean.

- **Median of Ratios per Patient:** For each patient, the REM AHI/NREM AHI ratio was calculated, followed by the median (ratio median, Equation (2)) of these values within each group. This is the median of the individual ratios and is less influenced by extreme values.

(2) $$\mathrm{ratio}\_\mathrm{meadian}=\mathrm{Mediana}(\frac{{\mathrm{AHI}\_\mathrm{REM}}_{i}}{{\mathrm{AHI}\_\mathrm{NREM}}_{i}})$$

Equation (2) ratio median.

- **Ratio of Means (delta-ratio-aggregate):** This is a synthetic group index, calculated by dividing the mean REM AHI by the mean NREM AHI within the same group, as seen in Equation (3).

(3) $$\mathrm{delta}\_\mathrm{ratio}\_\mathrm{aggregato}=\frac{\mathrm{mean}(\mathrm{AHI}\_\mathrm{REM})}{\mathrm{mean}(\mathrm{AHI}\_\mathrm{NREM})}$$

Equation (3) ratio of means.

**Figure 12:**
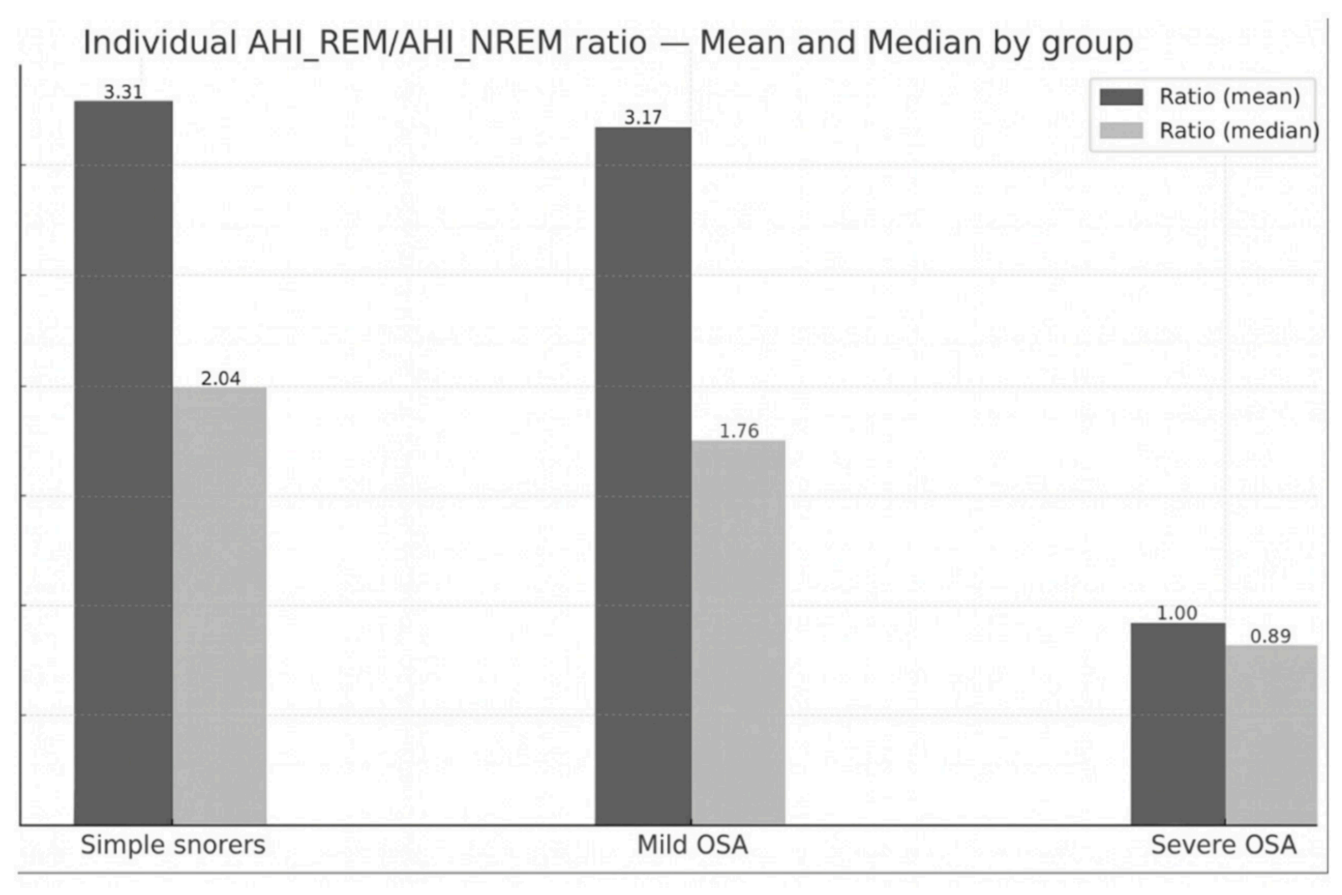
Ratio mean and ratio median.

The first two deltas described (ratio mean and ratio median) are shown in the graph below, Figure 12, illustrating their distribution across the various groups. As can be observed, there is a markedly higher REM/NREM delta in the simple snorers and mild OSA patient groups, with a normalization of this difference in the severe OSA group.

**Figure 13:**
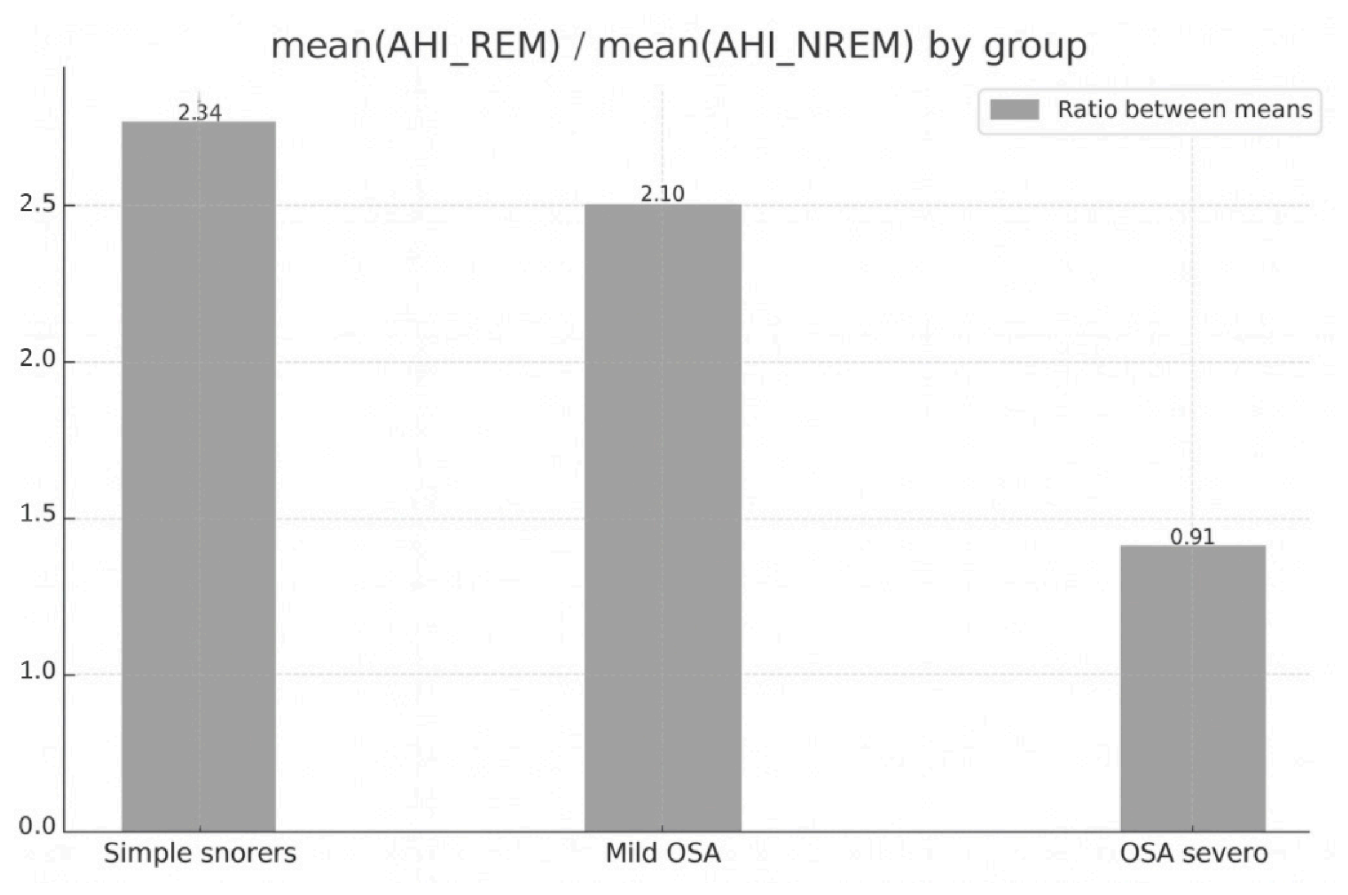
Ratio of means.

This difference is also observed for the disparity delta calculated using the ratio of means—the indicator we previously identified as more synthetic and descriptive, with lower clinical sensitivity. See the graph below, Figure 13:

In Table 12, the numerical correlates of the graphs are presented.

**Figure 14:**
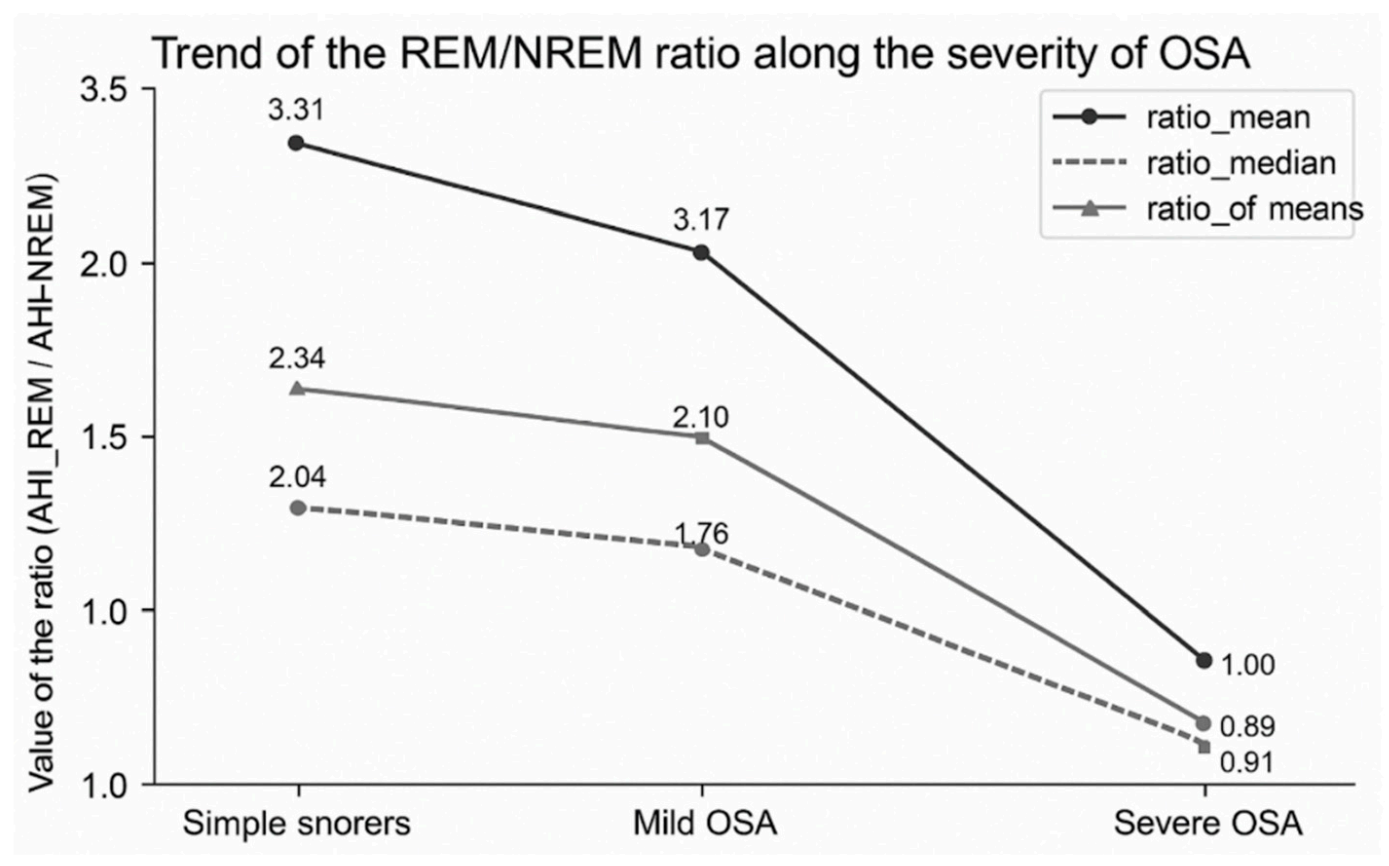
Trend of the REM/NREM ratio across OSA severity levels.

Finally, we present the behavior of the various calculated ratios across the different subgroups in Figure 14.

## 3. Discussion

**Figure 15:**
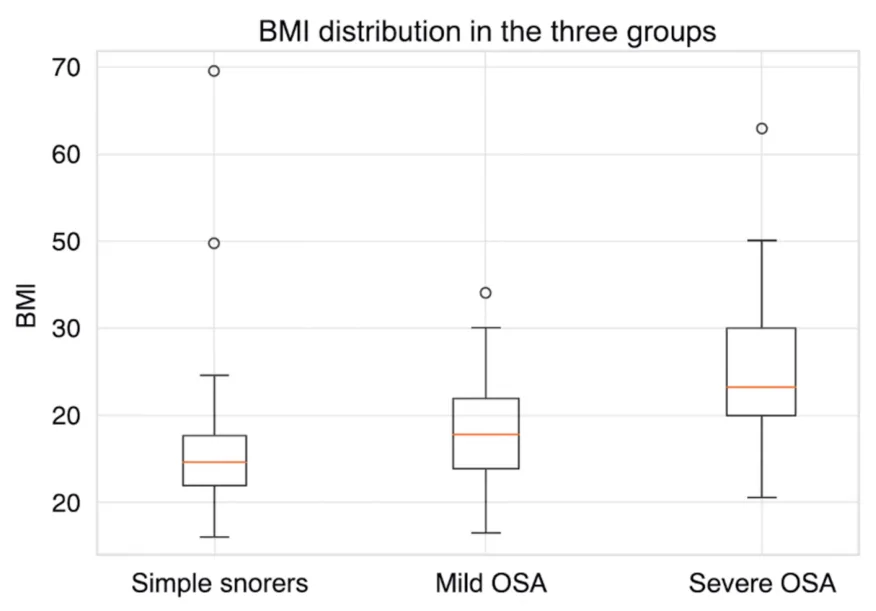
Distribution of BMI stratified by sleep-disordered breathing severity, to be interpreted in the context of anatomical contribution to the pathophysiological framework.

The primary finding of this study is the marked disparity in the REM/NREM AHI ratio across sleep-disordered breathing severity strata. In simple snorers and mild OSA patients, respiratory events cluster heavily in REM sleep (Ratiomean = 3.31 and 3.17, respectively). Conversely, in severe OSA, events occur pan-phasically, with AHI NREM matching or exceeding AHI REM (Ratiomean = 1.00; Ratiomedian = 0.89). This state-dependent shift provides an indirect clinical window into the functional balance between passive anatomical vulnerability and active neuromuscular compensation. In individuals with mild disease, pharyngeal collapsibility is mild to intermediate. During NREM sleep, preserved tonic and phasic neuromuscular recruitment of upper airway dilator muscles (principally the genioglossus and tensor veli palatini) is sufficient to maintain pharyngeal patency against collapsing forces. However, upon entering REM sleep, physiological motoneuron inhibition induces muscular atonia. Deprived of active neuromuscular compensation, the structurally vulnerable airway collapses, producing high REM AHI peaks that are otherwise effectively mitigated during NREM sleep. In contrast, in severe OSA, anatomical compromise is profound—further exacerbated by a significantly elevated BMI and excessive soft-tissue volume. Under these conditions, mechanical closing forces overwhelm active dilator recruitment during NREM sleep. Obstruction becomes structurally dominant and pan-phasic, abolishing the protective NREM benefit and driving the REM/NREM ratio toward unity or inversion. To support this anatomical component, BMI values across the three patient categories are shown below (Table 13, Figure 15).

Using the Wilcoxon and Kruskal–Wallis tests (Table 14), we assessed the direct difference between REM AHI and NREM AHI (i.e., whether the REM and NREM values within each group differ significantly). These raw data underpin the ratio calculations presented earlier. The results confirm that REM/NREM differences are indeed significant, and the Kruskal–Wallis test indicates that the ratios (from which ratio mean, ratio median, and ratio of means are derived) vary significantly between groups.

The Wilcoxon test revealed statistically significant differences in all groups analyzed: in simple snorers (*p* = 1.12 × 10^−4^) and mild OSA patients (*p* = 4.39 × 10^−4^), a clear REM predominance was observed, with REM AHI significantly higher than NREM AHI. In severe OSA patients (*p* = 2.18 × 10^−4^), a NREM predominance emerged, also statistically significant but weaker compared to the other groups. This reduced robustness (*p* just below 0.05) reflects greater internal heterogeneity in the severe OSA group: although a subgroup has very elevated NREM AHI, other patients show similar or inverted values between REM and NREM. Overall, the distribution remains significantly skewed toward higher NREM values, but with greater variability.

The Kruskal–Wallis test (H = 20.70, *p* = 3.19 × 10^−5^) confirmed the presence of significant differences across the three groups, demonstrating that the REM AHI/NREM AHI ratio systematically changes with disease severity.

It is interesting to note that, in the severe OSA group, the median REM AHI is slightly higher than the median NREM AHI, despite the statistically significant overall predominance of NREM. This apparent discrepancy reflects the group’s higher variability: while most subjects show similar values across the two sleep stages, a small number of patients with very high NREM values shifts the overall distribution.

In simple snorers and mild OSA patients, the REM AHI/NREM AHI ratio is clearly >1, indicating a strong REM predominance. This pattern suggests that, in mild cases, neuromuscular compensatory mechanisms are still partially effective during NREM sleep. In contrast, in severe OSA patients, the relationship reverses (ratio < 1), becoming NREM-predominant. Although the difference remains statistically significant, the lower level of significance and increased dispersion indicate that the disease, at this stage, has transcended functional differences in muscular response and is instead dominated by persistent anatomical factors. In other words, the loss of differentiation between sleep stages reflects a “pan-phasic” form of OSA, in which upper airway collapse is no longer modulated by sleep state but by the underlying anatomical severity.

REM predominance characterizes the milder subjects and gradually diminishes until reversing in severe cases, with statistical significance weakening as the disease becomes more uniformly expressed across all sleep phases. This pattern confirms a gradual transition from a phenotype more modifiable at the functional level to one primarily anatomical (structural collapse) along the continuum of OSA severity.

**Figure 16:**
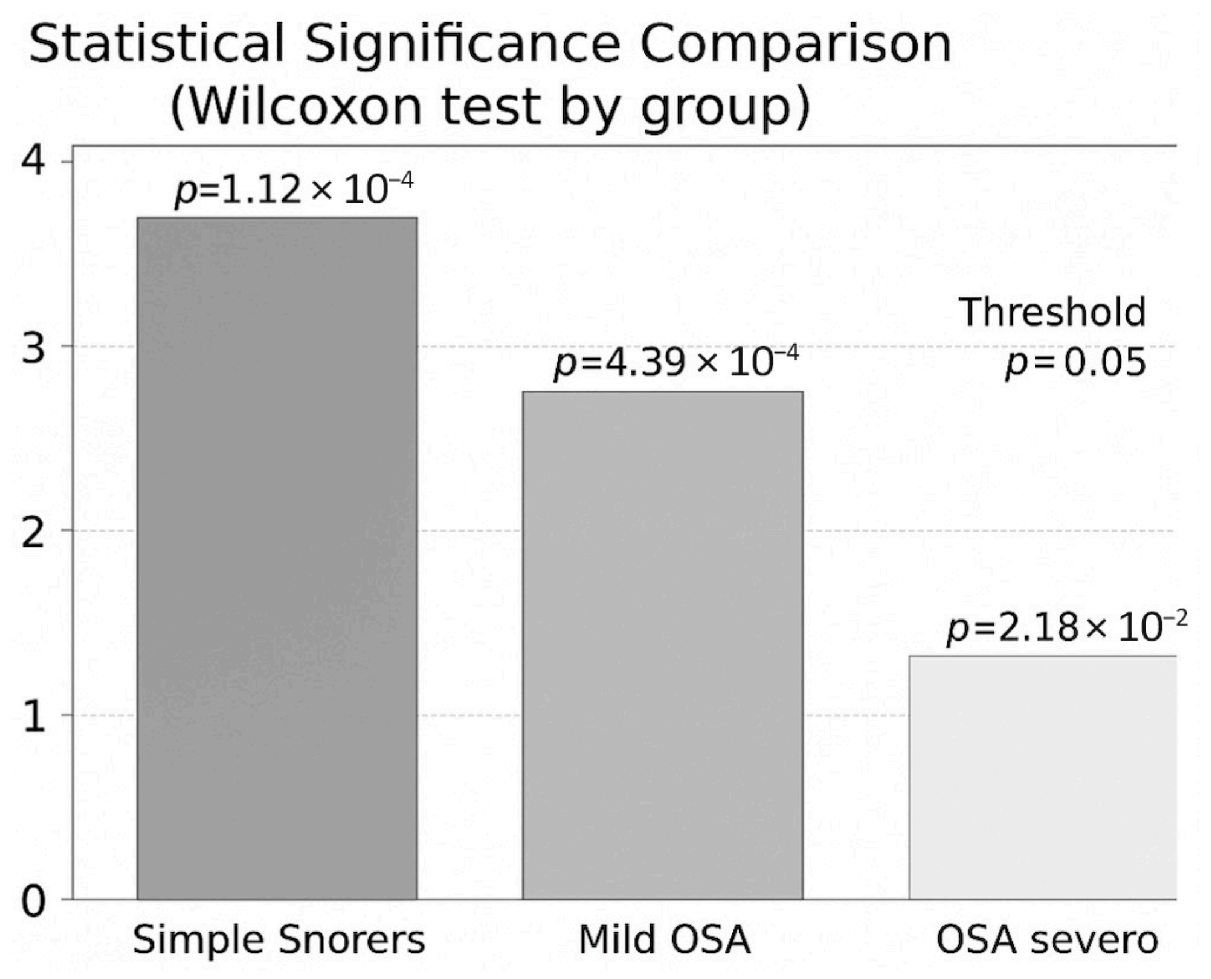
Comparison of statistical significance by group.

The summary graph below, in Figure 16, visually illustrates the concepts just discussed.

**Algorithmic Sleep Staging:** Sleep architecture was derived from the Nox BodySleep™ algorithm using actigraphy and respiratory plethysmography rather than gold-standard EEG/EMG polysomnography. While clinically validated, automated staging remains an indirect estimation, and misclassification of transitional epochs could introduce noise into stage-specific event frequencies.

**Spectrum-Selection Bias:** The deliberate a priori exclusion of patients with moderate OSA (AHI 15–30 events/h) created a non-contiguous severity spectrum. While useful for initial proof-of-concept contrast between phenotypic extremes, this design prevents us from determining whether the REM/NREM ratio declines linearly or undergoes an abrupt threshold drop across the disease continuum.

**Unmeasured Confounders:** Due to the anonymized database design, variables such as age, biological sex, precise supine versus non-supine time per stage, sedating or myorelaxant medications, and detailed cardiovascular comorbidities were unavailable. Body position, in particular, strongly influences NREM collapsibility and must be controlled in future prospective studies.

**Cross-Sectional Design:** Treatment outcomes were not evaluated, precluding any definitive conclusions regarding the predictive value of this metric for CPAP adherence or surgical success.

When interpreting NREM-predominant sleep-disordered breathing, the specific clinical context is crucial. In our severe cohort, NREM predominance co-segregated with high BMI (mean 32.51 kg/m^2^) and severe cumulative desaturations. This profile supports our interpretation that, in severely obese clinical populations, this pattern reflects an anatomically dominant phenotype where severe structural collapsibility completely overwhelms compensatory neuromuscular recruitment during NREM sleep. However, we acknowledge that in specific non-obese subsets, NREM predominance can be driven by different mechanisms. For instance, Thomas et al. [20] identified a distinct pathophysiological variant (CAP-dominant OSA) where NREM apneas were driven primarily by non-anatomical ventilatory control instability (elevated loop gain). Therefore, while the REM/NREM ratio remains a valuable and practical clinical surrogate for estimating the anatomical override of neuromuscular tone in typical obese patients, clinicians should remain alert to the possibility of ventilatory instability in specific phenotypes with prominent cyclic breathing.

Furthermore, the association observed in our severe cohort between NREM predominance and worse cumulative desaturation (CT90%) must not be interpreted causally. Individual REM apneas are typically longer and produce steeper desaturations. However, because NREM sleep constitutes approximately 75–80% of total sleep time, patients who lack neuromuscular compensation during NREM accumulate vast numbers of events across the night. The correlation with CT90% simply mirrors the aggregate disease burden and elevated BMI of severe pan-phasic collapse, rather than an isolated stage-specific insult. Within the PALM schema, identifying patients with preserved versus deficient muscle responsiveness remains an unmet clinical challenge. Drawing parallels with the physiological scoring model of Li et al. [16], tentative exploratory thresholds can be conceptualized: an AHIREM/AHINREM ≥ 1.30 ratio suggests a favorable neuromuscular trait (effective NREM compensation); a ratio between 0.80 and 1.30 represents an intermediate trait; and a ratio ≤ 0.80 denotes an anatomically overwhelmed phenotype. Because treatment outcomes were not longitudinally assessed in this cohort, these thresholds cannot currently guide therapeutic selection. Instead, they provide concrete hypotheses for prospective trials evaluating whether high-ratio patients demonstrate superior responses to non-CPAP modalities compared to pan-phasic phenotypes.

## 4. Materials and Methods

This exploratory retrospective study analyzed 207 overnight home respiratory polygraphy recordings from adult Italian patients evaluated for suspected sleep-disordered breathing between 2021 and 2023. The study used fully anonymized, pre-existing clinical records extracted from the Medicair Healthcare database (MedicAir Italia S.r.l. Via Monte Rosa, 61 20149 Milano, Italy). In accordance with national regulations and institutional policies, formal approval from an ethics committee was waived due to the retrospective and de-identified nature of the dataset. Written informed consent for data processing and scientific research purposes was previously obtained from all subjects at the time of their clinical evaluation.

**Figure 17:**
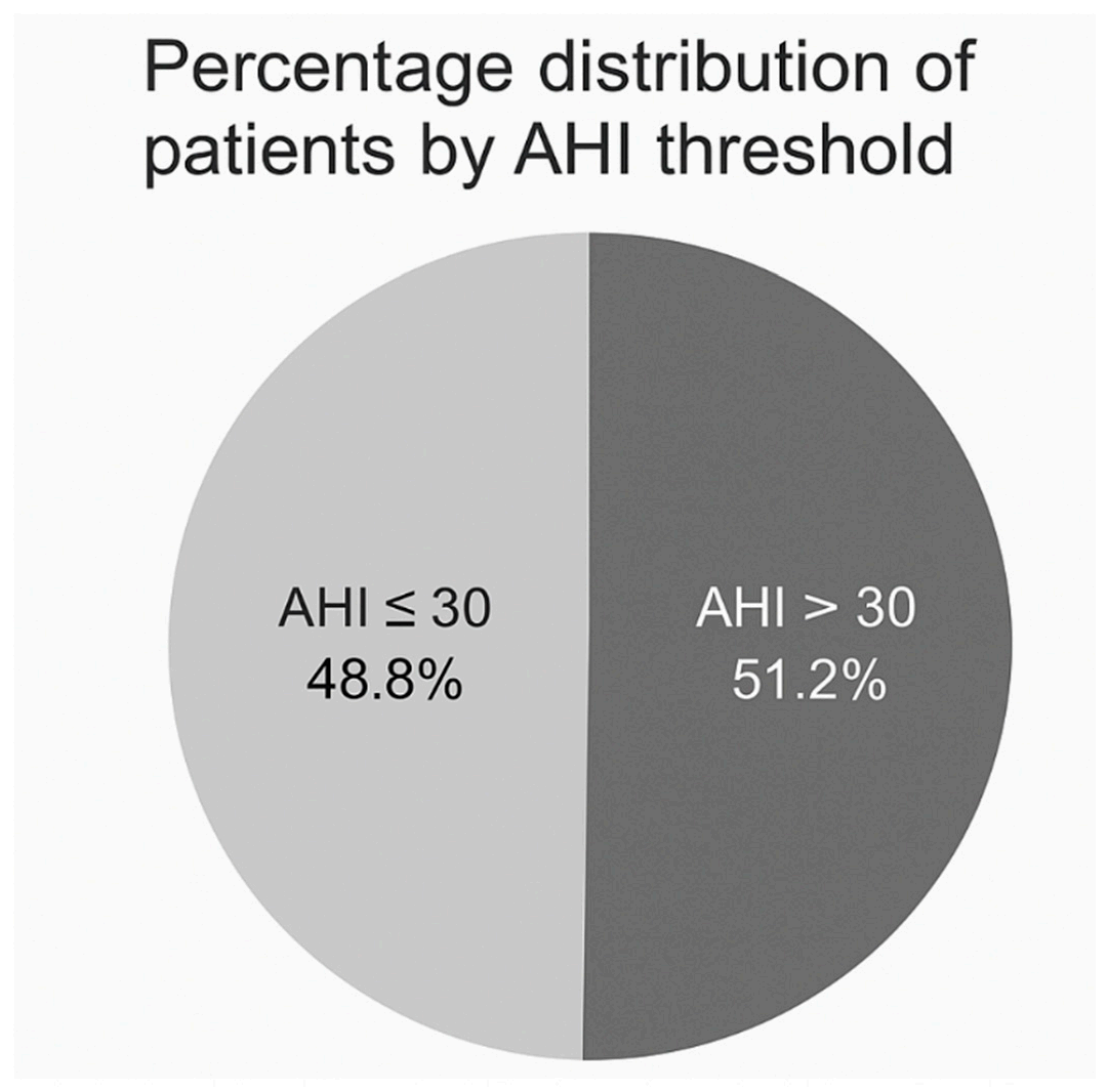
Percentage distribution of patients by AHI threshold = 30.

Eligible recordings were from adult patients (≥18 years) with technically adequate signal quality throughout the monitoring period, total recording time ≥ 6 h, and sufficient algorithm-estimated duration of both REM and NREM sleep. For this exploratory analysis, individuals with an overall Apnea–Hypopnea Index (AHI) between 15 and 30 events/h were intentionally excluded a priori. This non-contiguous sampling strategy was chosen for hypothesis-generating purposes to maximize phenotypic contrast between clearly separated severity strata at the extremes of the sleep-disordered breathing spectrum. The final analyzed cohort (n = 207) was stratified into (Figure 17):

- Simple snorers: AHI < 5 events/h (n = 48)
- Mild OSA: AHI 5–15 events/h (n = 53)
- Severe OSA: AHI > 30 events/h (n = 106)

All patients underwent home respiratory polygraphy using the Nox-T3 system (Nox Medical, Reykjavik, Iceland) equipped with dual-channel respiratory inductance plethysmography (RIP) belts, nasal pressure cannula, pulse oximetry, and body position/actigraphy sensors [22].

Because standard polygraphy lacks electroencephalographic (EEG) and electromyographic (EMG) channels, sleep architecture was estimated using the validated Nox BodySleep™ algorithm. This algorithm utilizes actigraphy, breathing dynamics, and plethysmographic pulse-wave features to classify 30 s epochs into Wake, REM sleep, and Non-REM sleep. During clinical validation against in-laboratory polysomnography, the algorithm demonstrated high accuracy (0.80) and substantial stage concordance (Cohen’s kappa = 0.70; F1 scores of 0.73 for REM and 0.82 for NREM epochs). Consequently, while this method provides a pragmatic and scalable clinical surrogate for sleep architecture, stage-specific metrics must be interpreted as indirect estimations rather than direct neurophysiological measurements.

Respiratory events were scored according to the American Academy of Sleep Medicine (AASM) criteria: apneas were defined as a ≥90% reduction in airflow for ≥10 s, and hypopneas as a ≥30% drop in airflow accompanied by ≥3% oxygen desaturation. For each patient, the following parameters were extracted: overall AHI, Apnea Index (AI), Hypopnea Index (HI), stage-specific AHI in REM (AHI REM) and NREM (AHI NREM), Body Mass Index (BMI), and conventional indices of cumulative nocturnal hypoxemia: percentage of recording time spent with SpO2 below 90% (CT90%) and cumulative minutes below 90% (T < 90 min).

### Statistical Analysis

Continuous variables were tested for normality using the Shapiro–Wilk test. Given the non-Gaussian distribution of stage-specific indices, data are presented as mean ± standard deviation (SD), median (interquartile range [IQR]), and ranges.

Within-group differences between AHI REM and AHI NREM were evaluated using the Wilcoxon signed-rank test and paired Student’s *t*-test. Effect sizes for Wilcoxon signed-rank tests were calculated using rank-biserial correlations (rrb) with 95% confidence intervals (CIs). Across-group differences in REM/NREM ratios were evaluated using the Kruskal–Wallis test, with epsilon-squared (ϵ2) calculated as the effect size.

Bivariable correlations between stage-specific metrics, BMI, and cumulative desaturation indices (CT90%, T < 90 min) were assessed using Pearson’s r and Spearman’s ρ. To control for false discovery rates across multiple correlation analyses, *p*-values were adjusted using the Benjamini–Hochberg procedure (critical threshold q < 0.05). Multivariable linear regression was conducted to determine whether NREM predominance was independently associated with cumulative hypoxemia (CT90%) after adjusting for BMI. Statistical analyses were executed using Python (v3.10; SciPy and Statsmodels libraries, London, UK) and IBM SPSS Statistics (v25.0), with significance set at a two-tailed *p* < 0.05.

## 5. Conclusions

In patients with non-severe OSA (simple snorers and mild OSA), REM AHI is on average higher than NREM AHI, suggesting relative neuromuscular protection during NREM sleep. Conversely, in subjects with severe OSA, NREM AHI prevails, accompanied by higher Apnea Index (AI) and significantly worse desaturation parameters (CT < 90% and T < 90 min). Considering the cohort as a whole, NREM predominance is associated with a globally more severe disease (higher AHI and AI, greater time spent in desaturation), whereas REM predominance characterizes milder and more variable presentations.

The REM/NREM delta (expressed either as an individual patient ratio or as a ratio of group means) progressively decreases with increasing disease severity, ultimately “normalizing” (approaching unity) in severe OSA. This pattern aligns with an anatomically dominant phenotype in which structural factors overshadow functional components.

These findings support the systematic use of REM/NREM AHI decomposition as a simple and readily available clinical surrogate for PALM-oriented profiling and personalized therapeutic decision-making. This approach should integrate—but not replace—total AHI, combining it with indices of hypoxemia and comorbidities.

## Figures and Tables

**Table 1:** Adapted from Li et al. [16]; 0 points indicates relatively good trait, 1 point indicates an intermediate trait and >2 points indicates a bad trait for passive anatomy, arousal threshold or upper airway muscle or control of breathing.

Parameter/Trait	0 Points	1 Point	2 Points	Standard β Values
Anatomy				
AHIREM, events/h	<20	20–50	>50	438
Arousals				
Fraction of events that were hypopnea, %	≥20	10–20	<10	595
Control of Breathing				
Loop gain	<0.6	0.6–1.0	>1.0	536
Central/mixed apnea index, events/h	<5	—	>5	655
Muscle Responsiveness				
AHIREM versus AHINREM	AHIREM < 30% AHINREM	30% AHINREM < AHIREM < 130% AHINREM	AHINREM > 130% AHIREM	582

It would therefore seem that REM-related OSA, occurring in a context devoid of muscular response, reveals, all else being equal, more severe pathophysiological values than its non-REM counterpart. To support this, the following studies are reported.

**Table 2:** Adapted from Brownell et al. [17].

Patient	SaO_2_ Threshold	Placebo (% Sleep Time)	Protriptyline, 6 mo (% Sleep Time)
1	<90%	46.60	30.10
	<85%	31.20	12.43
	<80%	17.97	3.55
2	<90%	24.18	11.47
	<85%	9.52	1.46
	<80%	2.94	0.54
3	<90%	79.22	50.23
	<85%	59.18	32.10
	<80%	46.57	19.31

**Table 3:** Adapted from Cartwright et al. [18].

Sleep Stage	Lateral Position	Supine Position	Supine − Lateral
NREM	12.27	61.25	48.98
REM	31.63	80.32	48.69
REM − NREM	19.36	19.07	—

**Table 4:** Values of mean AHI REM and NREM.

Group	AHI REM (Mean)	AHI NREM (Mean)	Difference (NREM − REM)
**AHI ≤ 30**	9.44	4.40	−5.03
**AHI > 30**	54.94	60.06	5.12

**Table 5:** Simple snorers Table.

Parameter	Count	Mean	Std	Min	25%	50%	75%	Max
AHI_REM	48	5.15	4.59	0.00	2.00	3.50	6.50	17.50
AHI_NREM	48	2.20	1.08	0.30	1.48	2.20	2.95	4.40

**Table 6:** Simple snorers.

Parameter (Simple Snorers, n = 48)	Mean	Median	Range (Min–Max)
AHI REM (events/h)	5.15	3.50	0.0–17.5
AHI NREM (events/h)	2.20	2.20	0.3–4.4
REM/NREM ratio	3.31	2.04	—

**Table 7:** Mild OSA Table.

Parameter	Count	Mean	Std	Min	25%	50%	75%	Max
AHI_REM	52	13.39	10.91	0.00	5.40	11.15	17.62	49.70
AHI_NREM	53	6.40	2.30	1.30	5.20	6.50	8.00	11.30

**Table 8:** Statistical correlations—mild OSA group.

Variable	Parameter	Spearman Rho	*p*-Value
AHI_REM	BMI	0.166	2.34 × 10^−1^
AHI_REM	Tlt90_perc	−0.027	8.46 × 10^−1^
AHI_REM	Tlt90_MIN	−0.004	9.77 × 10^−1^
AHI_NREM	BMI	−0.268	5.20 × 10^−2^
AHI_NREM	Tlt90_perc	0.194	1.63 × 10^−1^
AHI_NREM	Tlt90_MIN	0.164	2.40 × 10^−1^

**Table 9:** Severe OSA Table.

Parameter	Count	Mean	Std	Min	25%	50%	75%	Max
AHI_REM	105	54.94	21.65	0.00	40.50	56.20	70.50	96.40
AHI_NREM	106	60.06	24.85	23.00	42.52	54.15	76.12	154.90

**Table 10:** Severe OSA values.

Parameter (Severe OSA, n = 106)	Mean	Median	Range (Min–Max)	IQR (25th–75th)
AHI REM (events/h)	54.9	56.2	0.0–96.4	40.5–70.5
AHI NREM (events/h)	60.1	54.2	23.0–154.9	42.5–76.1
REM/NREM ratio	~1.0	0.89	0.0–2.9	0.67–1.23

**Table 11:** Spearman correlations between variables.

Parameter	Variable	Spearman Rho	*p*-Value
AHI_REM	BMI	0.524	9.42 × 10^9^
AHI_REM	T90perc	0.333	5.18 × 10^−4^
AHI_NREM	BMI	0.43	4.16 × 10^6^
AHI_NREM	T90perc	0.586	4.15 × 10^−11^

**Table 12:** Numerical correlates and synthesis.

Group	AHI REM Mean	AHI REM Median	AHI NREM Mean	AHI NREM Median	Ratio Mean	Ratio Median	Ratio of Means
Simple snorers	5.15	3.5	2.2	2.2	3.31	2.04	2.34
Mild OSA	13.39	11.15	6.36	6.35	3.17	1.76	2.10
Severe OSA	54.94	56.2	60.21	55.4	1.00	0.89	0.91

**Table 13:** Mean BMI in sub-groups.

Group	Mean BMI
Simple snorers	25.69
Mild OSA	28.30
Severe OSA	32.51

**Table 14:** Table of disparity deltas with statistical significance.

Group	Ratio Mean	Ratio Median	Ratio of Means	Wilcoxon_*p*	Direction	Significant (*p* < 0.05)
Simple snorers	3.31	2.04	2.34	1.12 × 10^−4^	REM > NREM	Yes
Mild OSA	3.17	1.76	2.10	4.39 × 10^−4^	REM > NREM	Yes
Severe OSA	1.00	0.89	0.91	2.18 × 10^−2^	NREM > REM	Yes

## Data Availability

The data presented in this study are available on request from the corresponding authors.
